# Lens growth and protein changes in the eastern grey kangaroo

**Published:** 2011-12-14

**Authors:** Robert C. Augusteyn

**Affiliations:** Vision CRC, Sydney, and Biochemistry Department, La Trobe University, Bundoora, Australia

## Abstract

**Purpose:**

Development in marsupials takes place predominantly ex utero while the young is attached to a nipple in the mother’s pouch, very different from that in other species. This study was undertaken to examine whether this affects lens growth and the production of lens proteins in kangaroos.

**Methods:**

Fresh lenses were obtained at official culls from eastern gray kangaroos (*Macropus giganteus*). Wet weights were recorded for all and protein contents were determined for one lens from each animal. Dry weights, after fixation were obtained for 20 lenses. Ages were determined using both molar progression and total lens protein content. Lenses were divided into concentric layers by controlled dissolution using phosphate buffered saline. Samples were taken for determination of protein contents and dry weights, which were then used to determine the age of the layer removed. Soluble crystallin distributions were determined by fractionation of the centrifuged extracts using HPLC-GPC and the polypeptide contents of both soluble and insoluble proteins were assessed by SDS–PAGE.

**Results:**

Lens growth is continuous from birth throughout adulthood and the increases in wet weight and fixed dry weight can be described with a single logistic growth functions for the whole life span. Three major crystallin classes, α-, β-, and γ-crystallins, were identified in the immature pouch-young animals aged around 60 days after birth. Adult lenses contain, in addition, the taxon-specific μ-crystallin. The proportions of these vary with the age of the lens tissue due to age related insolubilization as well as changes in the synthesis patterns. During early lactation (birth to 190 days), the α-, β-, and γ-crystallins represent 25, 53, and 20% of the total protein, respectively. After the pouch-young first releases the nipple (190 days), there is a rapid decrease in the production of γ-crystallins to around 5% of the total and a corresponding increase in μ-crystallin, from 0.5% to 15%. These changes were complete by the time the animal was fully weaned, around 1.5 years, and the final proportions of the 4 protein classes were maintained for the rest of life. The solubilities of α- and β-crystallins in the center of the lens decreased after age 5 years.

**Conclusions:**

Kangaroo lens growth is asymptotic, similar to that in most other species, even though most development of the young animal takes place ex utero. Changes in the patterns of lens protein synthesis in the kangaroo are similar to those observed in other species except for the large decrease in γ-crystallin and the matching increase in the marsupial-specific μ-crystallin, during late lactation.

## Introduction

Growth of the mammalian eye lens commences early in gestation and continues throughout life [1]. During this time, the lens accumulates high concentrations of a unique set of proteins, the crystallins, which generate the refractive properties needed for focusing images on the retina. The specific complement of proteins produced varies, not only between species, but also with ontogeny in some species. For example, human lenses produce substantial amounts of the γ-crystallins during prenatal life but their synthesis ceases at or just after birth [2] so that the adult lens cortex is devoid of γ-crystallins, other than the β_S_/γ_S_-crystallin polypeptide [3]. Bovine [4], pig [5], mouse [6], and rat [7] lenses also appear to experience changing patterns of protein synthesis, having low levels of γ-crystallins in the cortex compared with the nucleus. However, in these species there appears to be a gradual decrease in the level of γ-crystallins rather than an abrupt disappearance. In humans, the mode of lens growth changes, from asymptotic, prenatally, to linear for most of postnatal life, suggesting that birth, or an event associated with birth, affects the development of a functional human lens [8]. This does not appear to be the case for any other species for which data are available [1].

Lens growth in vertebrates takes place through a ubiquitous process in which new cells are laid down over pre-existing cells. This takes place within an elastic capsule so that no cells can be lost. Older cells are moved toward the center, lose their organelles and most metabolic capacity and become compacted. Since there is no turnover of cellular contents, the lens retains all of the proteins laid down throughout life, with the oldest in the center of the tissue and the most recently produced in the periphery. As the cells age, the proteins are gradually modified through deamidation, oxidation and truncation. By removing concentric shells of cells from a lens, it is possible to isolate cells and the proteins which were produced at different times in the lifetime of an animal. This permits evaluation of possible changes in synthesis patterns as well as the effects of prolonged existence [4].

Development in marsupials is very different from that in other mammals since it takes place predominantly ex utero [9]. In kangaroos, very immature young, only ~10 mm long and weighing ~0.8 g, are born after a short gestation period of 33–36 days, frequently interrupted by embryonic diapauses. They independently work their way up the mothers’ body to the pouch where they attach to a nipple and remain attached for about 190 days, while their bodies develop, before briefly venturing out. Around 45 days later, the young kangaroo, called a joey, starts to leave the pouch and must quickly find food supplies outside the pouch, learn to walk and keep warm by itself. However, it is usually allowed back in the pouch intermittently for about another 6 months and may continue to suckle.

Throughout pouch life, also referred to as the lactation phase of development, there are substantial changes in the composition of the marsupial mother’s milk [10]. The changes are particularly acute at the time when the young first detaches from the nipple, the transition from early to late lactation at around 190 days. Typically, milk produced during early lactation is dilute (9%–13% solids) and contains mainly protein and oligosaccharides. In late lactation, the milk becomes more concentrated (35%–40% solids) with increases in proteins and lipids while the carbohydrate content decreases. The changes in milk composition are accompanied by changes in the developing joey’s digestive tract and metabolic functions [11]. Subsequent weaning also leads to major digestive changes, some of which make the young animal temporarily susceptible to cataract formation if its diet includes galactose [12].

The unique ex utero development and the substantial changes in the young animal before it becomes independent raise the possibility that lens development may also be different from that in other mammals. The present study was undertaken to explore this possibility.

## Methods

No animal was sacrificed specifically for this project. All tissues used were byproducts from procedures performed by others at locations remote from the laboratory.

Eastern gray kangaroo (*Macropus giganteus*) eyes were obtained at officially approved culls conducted in Portland, Victoria, under Victorian Government guidelines. Eyes were collected within 1 h of death and cooled on ice. Lenses were removed no more than 12 h later and transported in individual containers, on ice, to the laboratory, where they were carefully examined for evidence of swelling and/or capsular rupture. Damaged lenses were discarded. Wet weights were recorded within 24 h after death.

Postnatal ages were initially estimated from molar progression and body dimensions, as described previously [13,14], and prenatal ages were determined from body dimension as described by Sharman et al. [15].

One lens from each animal was homogenized in phosphate buffered saline containing 0.03% sodium azide (PBSA). The protein content of the extract was determined with the Lowry method and used to determine animal age as described previously [14]. Good agreement was obtained with the age determined from molar progression. The combined extracts were centrifuged at 30,000× g for 20 min. The soluble kangaroo crystallins were isolated from the clarified extracts by gel filtration in PBSA on a 1.5×200 cm Biogel A 1.5M column (BioRad Laboratories, Hercules, CA) and their extinction coefficients (mg/A_280_) were determined from the relationship between the absorbance at 280 nm and protein concentration, determined with the Lowry method.

Twenty lenses were placed in 5% buffered formalin for 1 week, after which they were oven-dried at 80 °C until constant weight was achieved (2–3 weeks). The data were used to examine the relationship between lens wet and dry weights and age. Samples of the above PBSA extracts were also dried to determine the relationship between the total protein content and dry weight.

Eleven representative lenses, aged from 60 to 2,500 days after birth, were divided into concentric layers by controlled dissolution [4]. Protein contents and dry weights of the layer extracts were determined before and after centrifugation (10 min at 11,000× g) to remove insoluble proteins and the age of each layer was calculated from the accumulated protein and/or dry weight content as described previously [4].

Crystallin contents in the soluble fractions were examined by HPLC gel permeation chromatography (HPLC-GPC) in PBSA on Zorbax G250 and G450 columns (Dupont, Wilmington, DE) connected in series. The proportions of the soluble crystallins were calculated from the areas under the GPC peaks using the extinction coefficients of 0.8, 2.0, 2.0, and 1.0 /mg/ml at 280 nm determined for the kangaroo α-, β-, γ-, and μ-crystallins, respectively.

Polypeptides in whole lens extracts, layer extracts and the insoluble fractions, as well as proteins isolated by HPLC-GPC, were examined by SDS–PAGE and identified by comparison with those of the bovine lens. Proteins in the peaks separated by HPLC based gel permeation chromatography (HPLC-GPC) were first concentrated by precipitation with 5% TCA, washed with 1% TCA and dissolved in 0.1 M NaOH, before being examined with SDS–PAGE.

Polypeptide proportions were calculated from scans of the Coomassie Blue-stained gels, using Gene Tools (Synoptics, Cambridge, UK) after adjusting (×0.75) for the higher stain intensities of αB-crystallin and the β-crystallins due to their higher Lysine content [16]. Good agreement was then obtained between the proportions of the soluble crystallins determined from the HPLC fractionation and from SDS–PAGE of the same samples. By combining the data from the gel scans and the HPLC-GPC profiles, it was possible to identify and determine the distribution of the crystallins in different regions of the lens.

Identification of the 35 kDa band as μ-crystallin was confirmed by passing lens extracts through a Blue Sepharose CL-6B affinity column (Pharmacia, Uppsala, Sweden), eluting bound protein with 1 M NaCl and SDS–PAGE of the eluted protein. The molecular weight of the protein was estimated by comparison of the GPC-HPLC elution volume with those of known proteins.

## Results

### Lens weights

**Figure 1:**
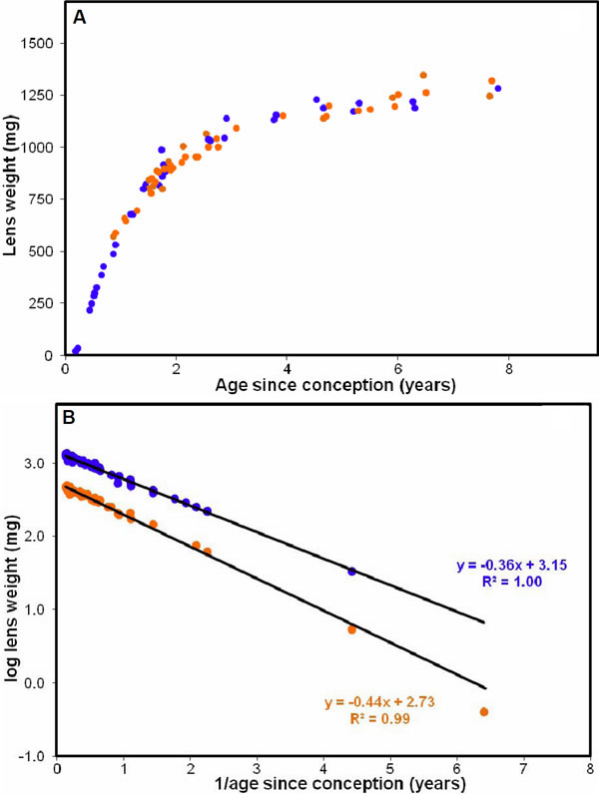
Age-related changes in eastern gray kangaroo lens weight. **A**: Changes in lens wet weight as a function of age since conception for males (blue filled circles) and females (orange filled circles). **B**: Logistic analysis of the changes in wet (blue filled circles) and dry (orange filled circles) weights for the combined male and female lenses.

Lenses were obtained from 63 eastern gray kangaroos, ranging from pouch young (PY), aged about 2 month after birth, to adults, near 8 years of age. Their wet weights ranged from 2.7 mg for the youngest animal to 1,250 mg for the oldest. The data are presented in Figure 1A as a function of age since conception (36 days before birth, assuming no diapause).

As noted previously for western gray kangaroos [14], lens wet weight increases asymptotically over the whole of the eastern gray’s lifespan and no differences can be observed between males and females, despite large differences in their bodyweights. Dry weights increase in a similar fashion (not shown). Logistic analyses of the combined male and female data (log weight versus 1/Age since conception; Figure 1B) yielded linear relationships for both wet and dry weights, with no suggestion of a transition to a different growth mode, as observed with human lenses [8]. The higher growth constant (slope) for dry weight (−0.44 years) compared with that for wet weight (−0.36 years) indicates that compaction takes place with increasing age. From the intercepts of the logistic plot, it may be calculated that the average dry mass concentration in the adult kangaroo lens approaches around 380 mg/ml.

The good fit of the dry weights to a logistic function (Figure 1B) was not unexpected since the final ages were based predominantly on protein contents. This indicates that lens dry weight could also be used for determining kangaroo age, as previously reported [17].

### Kangaroo crystallins

**Figure 2:**
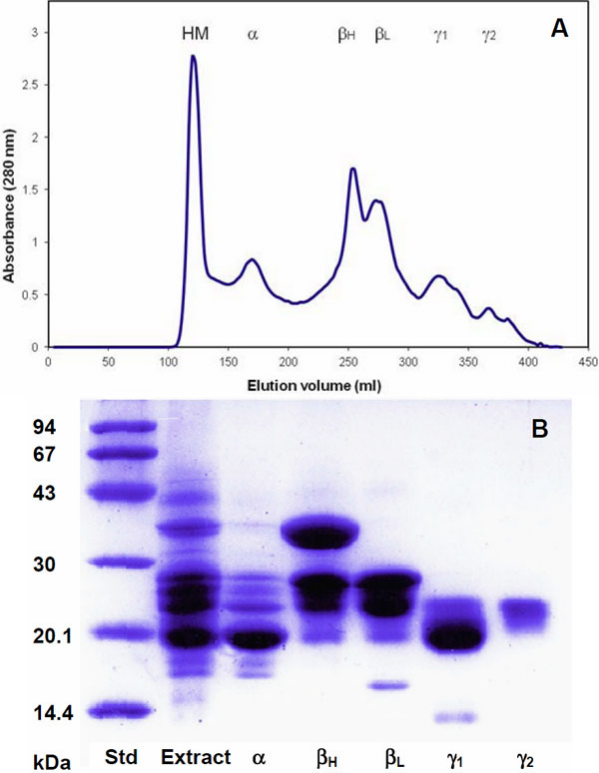
Fractionation of the water-soluble crystallins from a 2-year-old eastern gray kangaroo lens on Biogel A 1.5m. **A**: Elution profile and **B**: SDS–PAGE of Biogel peaks. Peaks were identified by comparison of the elution profile and SDS patterns with those of bovine lens proteins.

Gel filtration was used for a preliminary examination of the soluble crystallin contents in whole lens extracts. The profile (Figure 2A) and accompanying SDS gel (Figure 2B) indicate that, as with other mammals, the adult kangaroo lens contains predominantly α-, β_H_, β_L_, and- γ-crystallins, but other polypeptides can be seen.

The profiles included a substantial excluded peak, HM-crystallin, which increased with time when extracts were stored. This peak exhibited high light scatter and could be reduced with extended centrifugation, indicative of particulate matter. During storage, the insoluble protein content of the extracts also gradually increased, suggesting that the HM- crystallin complexes may represent a stage in the insolubilization process.

The α-crystallin peak (150–200 ml) is comprised mainly of αA-crystallin (20 kDa) with small amounts of contaminating β-crystallin polypeptides (22–28 kDa) from the adjoining peak and shorter polypeptides (16–18 kDa) which, from comparison with the proteins from other species, are probably truncation products derived from α-crystallin. Kangaroo αΒ-crystallin, which represents ~15% of the α-crystallin, co-migrates with the contaminating 22 kDa β-crystallin band.

Several bands, ranging in size from 14 to 35 kDa are observed in the β-crystallin peaks (200–300 ml). It is probable that the 14 kDa band is a truncation product. Of particular note is the very strong band of *M*_r_ ~35 kDa, associated with the β_H_-crystallin peak at 250 ml. This band bound to Blue Sepharose CL-6B, a characteristic of proteins containing the Rossmann dinucleotide fold [18] and was isolated as a 140 kDa tetramer. These properties indicate that it corresponds to the recruited protein, μ-crystallin which is unique to marsupials [19]. It appears as a doublet in the extract pattern, consistent with previous observations [19].

The two species of 20–22 kDa in the low molecular weight region (300–370 ml) correspond to the γ-crystallins. A band at around 10 kDa probably corresponds to the γ-crystallin degradation products, previously observed in human lenses [20].

### Crystallin polypeptide distribution

As there is no loss of cells or significant turnover of their contents, the lens retains all of the proteins produced throughout life, with the oldest in the center of the tissue and the youngest in the periphery. To examine the distribution of polypeptides at various stages of lens growth, 11 lenses, aged from 2 months to 7.7 years, were divided into concentric layers [4], each of which represented a different period in the growth of the lens. Generally, 5–7 layers were isolated but up to 13 were obtained with the larger lenses.

It was noted that the lenses contained a hard core which resisted dissolution and required homogenization to extract the proteins. The size and protein content of this core increased with the age of the lens from around 25 mg protein in the lenses younger than 1 year to an apparently constant 100 mg in all those older than 2 years. Insufficient data were available to determine whether this core was generated in a distinct growth phase, as observed in humans [8].

**Figure 3:**
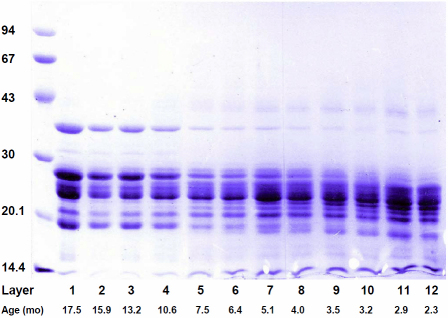
SDS–PAGE analysis of the soluble proteins in concentric layers from a 20-month-old eastern gray kangaroo. Sample numbers indicate the order in which the layers were removed from the lens, with layer 1 corresponding to the outer cortex + epithelium and layer 12 the most central region. The average age of each layer, in months, is indicated. Standard protein sizes (kDa) are indicated on the left.

The SDS–PAGE pattern for the soluble polypeptides in 12 concentric layers isolated from a single 20-month-old lens are shown in Figure 3. Very similar patterns were obtained with corresponding layers from other lenses.

Because of the changing complexity in the patterns, due to differences in the proteins synthesized at different ages, as well as age-related modifications, it was not possible to unequivocally identify all of the bands in the gel or accurately determine their proportions by scanning. However, it is clear that there are substantial differences in the polypeptide distributions.

In the outer layers (1–4), the patterns are relatively simple and dominated by species of approximately 35, 28, 26, 24, 22, and 20 kDa, corresponding to the major polypeptides of μ-, β-, and α-crystallins, as seen in Figure 2. Of particular note are the changes in μ-crystallin (35 kDa). Only traces (0.5%) can be seen in the central layers (8–12) but increasing amounts are found in the peripheral layers (1–4). Similarly, the 26–28 kDa β-crystallin band is a major constituent in the outer layers but absent from the central layers.

Closer to the center of the lens, the patterns become more complex and the polypeptide distributions shift to lower sizes. Increasing amounts of species around 14, 16, and 18 kDa are observed in the central layers. By comparison with Figure 2B, it may be seen that the larger two are truncation products derived from αΑ-crystallin (19–20 kDa) which decreases at the same time and has virtually disappeared by layer 9, while the smallest is derived from the β-crystallins.

### Soluble crystallins

HPLC-GPC was used to examine the distribution of soluble crystallins in the concentric layer extracts from 8 of the lenses. Because of the slow insolubilization of proteins, mentioned earlier, whenever possible, fractionations were performed within hours of the extraction. Despite this, several extracts still contained substantial amounts of insoluble proteins making some unsuitable for further analysis.

**Figure 4:**
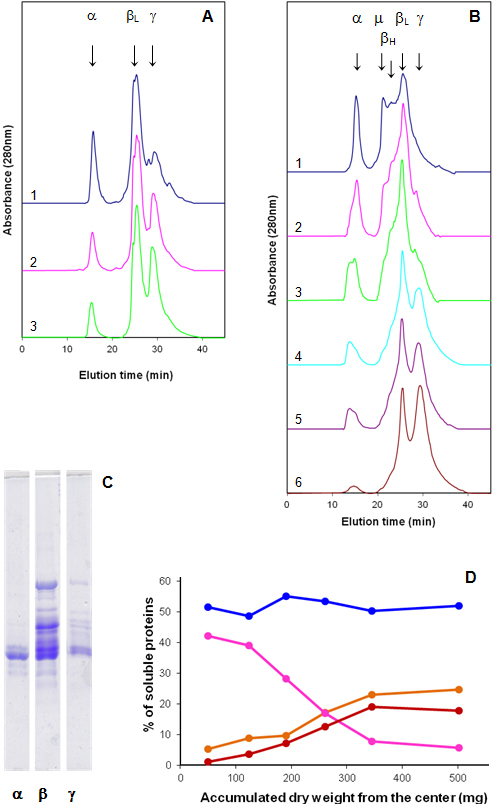
HPLC-GPC fractionation of the soluble proteins in concentric layers (numbered from the outside in) isolated from (**A**) a 130-day-old PY lens and (**B**), a 4-year-old eastern gray kangaroo lens. **C**: SDS–PAGE patterns of the three major peaks from the 4-year-old lens layers. **D**: Proportions of the soluble α- (orange), β- (blue), γ- (cyan), and μ- (red) crystallins from the 4-year-old lens layers (calculated from Figure 4B) as a function of the accumulated dry weight from the center.

Typical HPLC-GPC patterns for the lens layer extracts from a 130 days-old pouch-young (PY) and a 4 years-old kangaroo are shown in Figure 4A,B. Similar profiles were obtained for the corresponding layers from the other lenses.

SDS–PAGE of the TCA precipitated peaks from the inner and outer layers of the older lens (Figure 4C) indicated that the first peak, eluting at 15 min, contained only α-crystallin. Scanning of the gel indicated that the αA/αB polypeptide ratio, corrected for differential staining, was ~9, consistent with previous observations [21,22]. The second major peak, eluting between 20 and 28 min, consisted of polypeptides ranging in size from 18 to 35 kDa. In the older lens, the central peak comprised 3 major components, μ-, β_H_-, and β_L_-crystallins, The third major peak (30 min) contained the two γ-crystallins of 20 and 21 kDa, together with small amounts of β-crystallins.

The HPLC-GPC profile for the 130 day PY lens (Figure 4A) is simple, comprising the 3 major mammalian protein groups, α-, β-, and γ-crystallins. β_H_- and μ-crystallins are virtually absent. However, even at this early age, differences can be seen across the lens. The α-crystallin peak decreases from around 30% of the total soluble protein at the outside to 19% at the center. By contrast, the γ-crystallin peak increases from 17 to 34%. The β-crystallins appear to remain relatively constant around 50%.

Similar, but more pronounced, changes can be seen across the 4-year-old adult lens (Figure 4B). Substantial decreases are evident in the μ-, β_H_-, and α-crystallin components, toward the center, so that the nuclear (layer 6) soluble proteins consist predominantly of β_L_- and γ-crystallins. The proportions of the various soluble proteins in the 4 year old lens layers, calculated from the profile in Figure 4B, are plotted in Figure 4D as a function of accumulated mg of dry weight from the center, i.e., from the oldest to the youngest regions of the lens. The proportion of α-crystallin ranges from less than 5% in the center to nearly 25% of the soluble protein in the peripheral layer; μ-crystallin increases from <1% to 18%; the γ-crystallins decrease from almost 40% to near 6%; while the β-crystallins appears to be relatively constant at 52%. The proportions in corresponding layers from the other lenses were very similar. The changes observed are consistent with the changes in polypeptide patterns shown in Figure 3.

**Figure 5:**
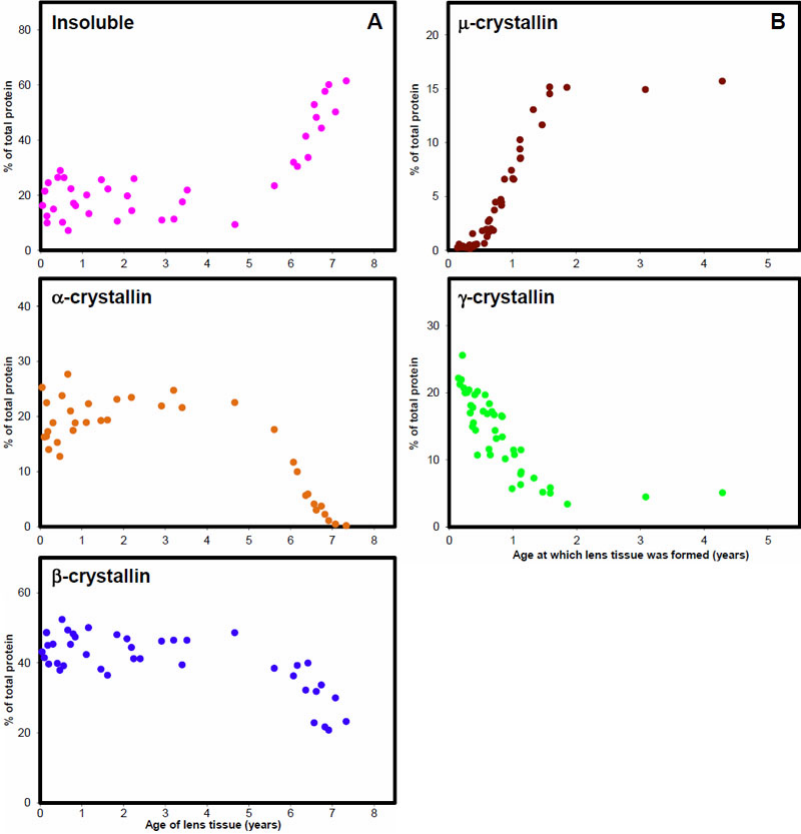
Crystallin proportions in the kangaroo lens. **A**: Soluble α- and β-crystallins and insoluble proteins as a function of age since conception. **B**: γ- and μ-crystallins as a function of age at which the lens tissue was formed.

It should be noted that the proportions quoted above are representative of the soluble protein fraction only and must be adjusted to take into account the insoluble protein content of each layer. This has been done in Figure 5 which summarizes the proportions of soluble crystallins and insoluble proteins in 49 layers from 8 lenses.

The distributions of the proteins can be presented in two ways, as a function of the age at which they were produced (inside to outside of lens) or as a function of the age of the tissue, i.e., the time since they were produced (outside to inside of lens). Different information is obtained from each.

Alterations in protein solubility are best revealed by plots of their proportions against age of the tissue (layer) while changes in their production become evident when proportions are plotted against the age of the animal when the layer was produced. In some parts of the lens both changes in solubility and synthesis patterns are observed but these can generally be distinguished by comparing the two plots. When such comparisons were performed with the present set of data, it was clear that the proportions of α- and β-crystallins and the insoluble proteins were related to the age of the tissue but γ- and μ-crystallins levels depended on the age at which the layer was produced.

The age-related alterations in α- and β-crystallins and the insoluble proteins are shown in Figure 5A. Most of the protein (80%–90%) in the youngest, outer lens layers is soluble but in the older center up to 70% is insoluble. Figure 5A suggests that the increase starts when the tissues are around 4–5 years old. Some of the insolubilization may be attributed to precipitation during storage, as suggested by the experience with one lens where up to 60% of the protein became insoluble during overnight storage of the layer extracts at 5 °C. The soluble crystallin data from this lens were discarded. It is probable that the low insoluble protein content at 5 years (9%) is due to some sample loss during the isolation. Nevertheless, it would appear that the older proteins in the center of the lens are less soluble when the tissue is disrupted.

In the young periphery of the lens, soluble α-crystallin accounts for 20%–25% of the total protein. In regions older than 4–5 years, it rapidly decreases and is virtually gone (<1%) by 7 years. Similarly, soluble β-crystallins are highest (40%–45%) in the youngest tissues and after age 4–5 years decrease rapidly to around 20%. The combined decreases in the soluble α- and β-crystallins appear to closely match the increase in the insoluble protein. When plotted as a function of the age at which the tissue was produced, the data for these 3 groups were randomly distributed.

Different conclusions are reached from a consideration of the data for the μ- and γ-crystallins. When plotted as a function of age of the tissue the data are randomly distributed. However, when examined as a function of the age at which they were produced (Figure 5B), clear tends emerge. Very early in life, very little μ-crystallin is produced (0.3%–0.4%) but from ~160 days after birth, production of the protein rapidly increases up to a maximum of 15% of the total protein by around 1.5 years, after which it appears to remain constant. High levels of γ-crystallin (20%) are produced early in life. However, apparently also starting around 160 days, there is a rapid decrease to 4%–5% by age 1.5 years, remaining at this level thereafter. It would appear that these changes are correlated since the sum of μ- and γ-crystallins is relatively constant throughout life at 18.3 ±2.6% of the total proteins in any part of the lens.

### Insoluble proteins

**Figure 6:**
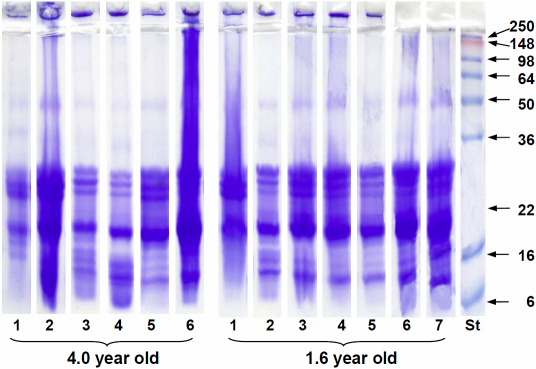
SDS PAGE of eastern gray kangaroo lens insoluble proteins in 7 concentric layers from a 1.6-year-old lens and 6 layers from a 4-year-old lens. Layers are numbered in order of their removal from the lens so that layer 1 is the outermost from each lens and layer 6 (4-year old) or 7 (1.6-year old) is the innermost. Standard protein sizes (kDa) are indicated on the right.

Although attempts were made to minimize the insolubilization of the protein, Figure 5A indicates that many of the layers contained significant amounts of insoluble protein and, in some cases, more than similarly aged layers from other lenses. Since this complicates the interpretation of possible age-related changes, SDS–PAGE was used to examine which polypeptides became insoluble. The patterns obtained with the insoluble proteins in all 7 layers obtained from a 1.6-year-old lens and all 6 layers from a 4-year-old lens are shown in Figure 6. Very similar patterns were obtained with the insoluble proteins from other lenses.

Apart from the protein which did not enter the gel (3%–6% of the total Coomassie staining), presumably membrane and cytoskeletal components, the predominant (>95%) insoluble species in all layers appear to be α- and β-crystallin polypeptides. In addition, all layers except the outermost, contained the truncated 14, 16, and 18 kDa species. No significant amount of insoluble μ-crystallin was observed in any layer. It was not possible to distinguish γ-crystallin from the other polypeptides but it was concluded that, given soluble γ-crystallin decreased in the outer layers, it was unlikely that significant amounts would be present in the insoluble fraction. Although there are changes in the proportions of individual β-crystallin polypeptides (22–30 kDa) and differences in the amounts of the degraded species (14–18 kDa), the relative amounts of α- and β-crystallin polypeptides (including truncated species) were remarkably constant, averaging 32.5±1.6 and 62.8±2.6%, respectively, of the total Coomassie staining in all layers examined, regardless of the total amount of insoluble protein. After adjusting for the differences in stain intensity, the proportions became 39 and 56% of the insoluble protein, respectively.

**Figure 7:**
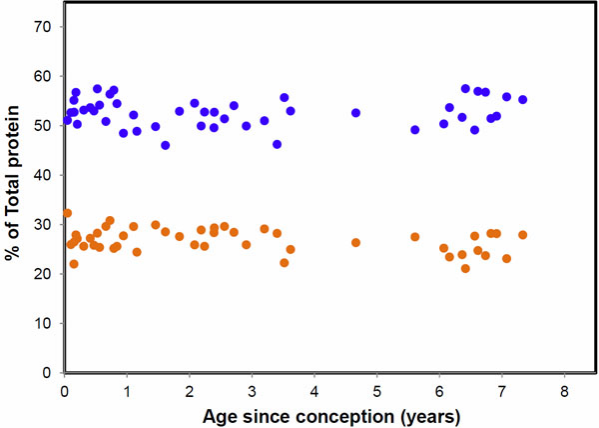
The proportions of soluble plus insoluble α- (orange filled circles) and β-(blue filled circles) crystallins as a function of age since conception.

The proportions of the combined soluble and insoluble α- and β-crystallins are presented in Figure 7. It is clear from these data that the amounts of the two proteins synthesized at different stages of life are constant, with α-crystallin representing 25±2.4% and β-crystallin, 54±2.7%.

## Discussion

This study was undertaken to explore the idea that marsupial lens growth might be different from that in other species because development of the embryo takes place predominantly ex utero. This turned out not to be the case. Nevertheless several interesting observations were made.

It was noted that fixed lens dry weight, increased asymptotically, similar to the wet weight and protein content, making it suitable for use in age determination. Methods have previously been presented for estimating kangaroo ages from lens protein content [14] and from (fixed) dry weight [17]. Neither is completely satisfactory. The protein based method requires access to laboratory facilities, not always readily available. The dry weight method, which is commonly used for determining ages of animals in field studies, is far more convenient. However, the previous study [17] did not take into account that lens growth is asymptotic [8,14] and consequently, the simple algorithm presented (log Age=Constant + k_*_ Lens dry weight) overestimates the ages of young kangaroos and underestimates those of older animals.

The equation for the linearized logistic fit of the present data can be rearranged to yield a simple relationship for calculating kangaroo age from lens dry weight, Age=0.44/(2.73-Log Lens dry wt), where age is in years since conception (years since birth +0.1, ignoring diapause) and Lens dry weight is in mg. Analysis of lens dry weights using this equation will provide a reasonable estimate of the age of eastern gray kangaroos over the whole of the life span.

Because of the unique growth mode, in which no cells or their contents are lost, the lens retains all of the proteins laid down throughout life. Those in the lens center are the oldest, having been laid down early in gestation. Those further from the center are progressively younger. Thus, comparison of protein distributions in concentric layers can give valuable information on the patterns of protein synthesis at different stages of growth and development.

In old lenses, such data may be complicated by the effects of prolonged existence on the proteins. This includes protein aggregation and insolubilization which increases with age and therefore, will be greater in the central regions of the lens [23,24]. Substantial amounts of insoluble protein were observed in this study. Some were generated during storage, a characteristic of kangaroo lens extracts, but most were present in the fresh extracts. The amount of insoluble protein observed in the current study has little relevance to the in vivo situation, since all lenses were transparent. Nevertheless, it must reflect some differences in the protein properties. Thus, the proteins more than 5 years old from the hard core of old lenses are less soluble than those in the periphery. As has been noted previously [24], lens crystallin insolubilization does not appear to be related to protein modification. With the exception of the peripheral layer, no differences were observed in the truncated polypeptide contents of the soluble and insoluble proteins from the various lens layers. This suggests truncation takes place not long after the proteins have been produced and may be required for the packing of the proteins and compaction of fiber cells.

The crystallin contents of the kangaroo lens were similar to those of other species, consisting predominantly of α- β-, and γ-crystallins in the young animal [25]. However, the α-crystallins accounted for a smaller proportion (25%) and the β-crystallins a larger proportion (54%) of the total than those found in other species (40–50 and 30%–40%, respectively) [25]. It may be that the α-crystallin content of the lens is higher in the more recently evolved species to improve the chaperone capacity and prolong transparency. The proportions of these two crystallins appear to be constant throughout life but both become insoluble, at about the same rate, when the lens is disrupted and account for >95% of the insoluble protein. This differs from bovine and human lenses where α-crystallin becomes insoluble long before the β-crystallins and accounts for >80% of the insoluble protein in all but the oldest region of old lenses [4,23].

For the proteins which do not become insoluble, as found for μ- and γ-crystallins, it is possible to examine their ontogeny. The decrease observed in the kangaroo γ-crystallin distribution is similar to that observed in bovine lenses [4], but appears to take place more rapidly. Around 200 days after birth, γ-crystallin synthesis starts to decrease and levels off at near 5% by 1.5 years of age. By contrast, in other species, such as the rat and trevally [26], substantial production of these proteins persists throughout life. In some ways, the kangaroo pattern resembles that observed in the human lens where γ-crystallin synthesis takes place only in the prenatal growth phase which concludes very soon after birth [8]. Thus, like in the human lens, kangaroo γ-crystallin is mainly limited to the central or nuclear region of the lens.

A distinguishing feature of marsupial lenses is the presence of the unique, recruited protein, μ-crystallin [19]. The present study has revealed that this protein is expressed at around 0.5% of the total protein until about 200 days after birth (100 mg dry weight) when its synthesis is accelerated until it represents ~15% of the total protein by 1.5 years. This level is maintained throughout the rest of life.

The surge in μ-crystallin production occurs very close to the time of eye opening at the end of the Cecal period (~190 days) and when the joey first releases the teat and views the world outside of the pouch. As mentioned earlier, this is also the time when γ-crystallin synthesis starts to decrease. It would appear that the amounts of the two proteins are inversely related, maintaining a constant 18%–20% of the total. What the signal for these changes may be remains to be established. Perhaps it may be first exposure of the retina to light following eye opening.

The reason for the changing protein synthesis patterns are not obvious. γ-Crystallin concentration vary widely, not only across the lens, but also between species, suggesting that they play more than a space filling role. It may be that γ-crystallins, which exhibit attractive interactions, actively promote closer packing of the crystallins to generate high refractive index tissues. Thus, they may be responsible for the compaction and hardening/stiffening of lens tissue [24]. In rodents and fish, high levels of the protein are found throughout the lens and are associated with a ‘rock-like’ consistency; in humans, pigs and cows, the nucleus is softer but hard relative to the cortex, which contains very little or no γ-crystallin; while bird and reptile lenses, which lack the protein, altogether, are very soft. Observations during the present study suggested that, like the human lens, the adult kangaroo lens may also have a distinct hard nucleus throughout which the highest concentration (20%) of γ-crystallin is found. The mass of this nucleus (100 mg dry weight in adult animals) suggests it was laid down during the Cecal period. It may be that a hard nucleus and a soft cortex are reflections of high and low refractive index tissues, required to satisfy the particular visual requirements of an animal. However, there does not seem to be a relationship between the γ-crystallin content and the distribution of refractive index. In humans, the nucleus has a gradient of γ-crystallin concentration but a refractive index plateau which increases up to a maximum by around age 60 years. Other species have continuously increasing γ-crystallin concentrations across the whole lens and RI gradients which peak in the center of the lens. The shape of the RI profile in kangaroos is not known.

Several functions have been proposed for μ-crystallin in the lens. It has been observed that μ-crystallin has thyroid hormone (T3) binding properties suggesting that it may play a role in regulating glucose metabolism in the cortex of the kangaroo lens by modulating the availability of thyroid hormone [27]. This may be related to the change in milk carbohydrate content during late lactation. However, it is hard to understand why the lens, which has very low metabolic activity, should need the high thyroid-binding capacity provided by a protein present at a concentration of around 2 mM and representing 15% of the total peripheral protein.

The μ-crystallin T3 binding activity is stimulated by NADPH [27]. Thus, like many other recruited crystallins, μ-crystallin binds NADP/NADPH. It has been suggested that this property of recruited crystallins may be important in providing a UV filter for the lens [28]. Such a suggestion would be consistent with the increased production of the protein in the kangaroo lens, immediately the joey is first exposed to light.

It has recently been shown that μ-crystallin has ketimine reductase activity which is strongly inhibited by T3. It was suggested that this activity could be important in amino acid metabolism and play a role in the regulation of neurotransmittor/neuromodulator levels in the brain [29]. However, such a role seems unlikely in the lens. More likely, the protein has been recruited for use as a lens protein because of some other, as yet unidentified property. It is interesting that it appears to replace γ-crystallin, suggesting it may play a role in the regulating lens hardness/hydration.

In conclusion, the current study has shown that, although humans and kangaroos are a long way apart on the evolutionary tree and their embryonic development is quite different, growth of the lens and changes in its protein contents are remarkably similar.
